# Comparison of macular changes and visual outcomes between femtosecond laser-assisted cataract surgery and conventional phacoemulsification surgery for high myopic cataract patients

**DOI:** 10.1186/s12886-024-03479-6

**Published:** 2024-05-15

**Authors:** Xuemei Liang, Shinan Luo, Kaiyu Deng, Li Li

**Affiliations:** 1 Department of Ophthalmology, Aier Eye Hospital, Jinan University, No. 191, Huanshi Middle Road, Yuexiu District, Guangzhou, Guangdong 510071 PR China; 2 Department of Ophthalmology, Nanning Aier Eye Hospital, Nanning, China

**Keywords:** Central foveal thickness, Choroidal thickness, Best-corrected visual acuity, Conventional phacoemulsification surgery, Femtosecond laser-assisted cataract surgery

## Abstract

**Background:**

To evaluate differences in log MAR best-corrected visual acuity (BCVA) improvement and postoperative central foveal thickness (CFT) and choroidal thickness (CT) changes between conventional phacoemulsification surgery (CPS) and femtosecond laser-assisted cataract surgery (FLACS) for high-myopia cataracts.

**Methods:**

This was a retrospective and observational study. One hundred and two eyes of 102 patients with high-myopia cataracts were examined. CPS was performed in 54 eyes, and FLACS was performed in 48 eyes. All eyes underwent logMAR BCVA, CFT and CT of three different sectors preoperatively and one week and six months postoperatively.

**Results:**

The logMAR BCVA improved significantly after surgery in both groups (both *P* < 0.001), but no difference was observed in BCVA improvement between the groups (*P* = 0.554). Moreover, no significant differences were reflected in the changes in CFT, nasal 1 mm CT or temporal 1 mm CT between the two groups, and only subfoveal choroidal thickness (SFCT) in the CPS group decreased significantly compared with that in the FLACS group at any postoperative time (*P* = 0.003 and 0.026). AL, preoperative logMAR BCVA, and CT of the three regions exhibited a notable correlation with postoperative BCVA (all *P* < 0.05) according to univariate logistic regression analysis. However, only the AL, preoperative logMAR BCVA and SFCT remained significant in the multivariate model. Postoperative logMAR BCVA revealed a positive correlation with AL and preoperative logMAR BCVA but a negative correlation with SFCT.

**Conclusions:**

FLACS was not superior to CPS in improving BCVA but had less impact on SFCT in the treatment of high-myopia cataracts. Eyes with a longer AL, worse preoperative logMAR BCVA and thinner SFCT had a high risk of worse postoperative BCVA.

## Introduction

Myopia is one of the most common eye diseases globally, and its prevalence remains higher in Asia (60%) than in Europe (40%) [1]. High myopia is defined as a spherical equivalent (SE) ≤ − 6.00 diopters (D) or axial length (AL) ≥ 26 mm [2]. The prevalence of myopia and high myopia (HM) has increased significantly worldwide, affecting an estimated 34% and 5.4%, respectively, of the world population in 2020 [3]. Pathologic myopia (PM) is usually defined as HM combined with typical fundus lesions, including chorioretinal atrophy, lacquer cracks, staphyloma, and vitreomacular traction [4]. Eyes with high myopia tend to develop earlier-onset and more progressive nuclear cataracts (a three-to-fivefold increase in risk) and posterior capsular cataracts (a 30% increase in risk) [5, 6]. Currently, surgery is the only effective therapeutic method for cataract treatment. High myopia-induced cataracts account for 30% of all cataract surgeries in tertiary hospitals [7].

Modern cataract surgery has been confirmed to be safe and effective in treating highly myopic cataracts. However, fluctuations in intraocular pressure (IOP) and ultrasound energy during phacoemulsification may impair optic nerve perfusion, the retina or the choroid [8, 9]. Moreover, an earlier study revealed that high myopia was frequently associated with certain structural changes, including retinal and choroidal thickness thinning [10]. Phacoemulsification may cause or accelerate the progression of myopic macular pathologies due to the development of macular traction and oedema [11, 12]. With the advent and growing use of optical coherence tomography (OCT), more subtle macular morphology and structural changes have been identified in high myopia patients after cataract surgery [13–15].

While extensive comparative research has been conducted on retinal and choroidal structural alterations in conventional phacoemulsification surgery (CPS) and femtosecond laser-assisted cataract surgery (FLACS), few investigations have focused on evaluating these changes in high-myopia cataracts. Therefore, the purpose of this study was to assess the effects of myopia on chorioretinal thickness after cataract surgery, compare the changes between the CPS and FLACS by SD-OCT, and analyse the risk factors for postoperative vision. We aimed to assist surgeons in choosing the optimal surgical method to minimize the adverse effects of surgery on the retina and choroid.

## Materials and methods

This retrospective observational clinical study involved patients who were diagnosed with cataracts accompanied by nonpathologic high myopia (AL ≥ 26 mm) at Nanning Aier Eye Hospital from July 2020 to July 2023. The operated eyes were divided into two groups based on the surgical methods applied. The following inclusion criteria were used: no other ophthalmic or systemic diseases except for high myopia and cataracts and an age of at least 18 years. The following exclusion criteria were used for CT measurements: (1) no morphological changes associated with myopia, such as retinal detachment, geographic atrophy, epiretinal membrane (ERM), or retinoschisis (RS), which involves the macula; lamellar macular hole (LHM), full-thickness macular hole (FHM), or choroidal neovascularization (CNV); (2) a history of diabetes mellitus, glaucoma, retinal vascular occlusion, macular diseases, retinal detachment, or uveitis; (3) previous trauma or ocular surgery; and (4) inability to cooperate during OCT measurements. All patients signed written informed consent forms. In patients who underwent surgery in both eyes, only the first operated eye was included.

### Examinations and operation

Patients underwent routine ocular examinations before the operation and then at one week and six months postoperatively, including BCVA measurements using decimal charts, which were converted to logMAR for reporting purposes. Counting fingers represented a log MAR of 2.0, hand movements represented a log MAR of 2.3, light perception represented a log MAR of 2.7, and no light perception represented a log MAR of 3.0 [16]. The IOP was measured with a CT80A noncontact tonometer (Topcon, Japan). Slit-lamp biomicroscopy, wide-angle scanning laser ophthalmoscopy (SLO) (panoramic 200Tx, OPTOS, UK), and optical coherence tomography (OCT) (Spectralis-OCT, Heidelberg Engineering, Germany) were also performed. Axial length was measured using an IOL Master 700 (Carl Zeiss AG, Oberkochen, Germany) before the operation.

**Fig. 1:**
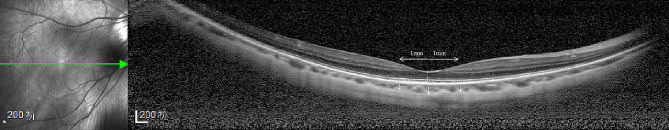
Image presenting the technique for retinal and choroidal thickness measurement by SD-OCT

OCT images were obtained with Spectralis SD‑OCT using the retest function at baseline and at one week and six months after the operation by two experienced specialized doctors (SN.L. and KY.D.). Images of each section were obtained via eye tracking, and an average of 80 b-scans were utilized to improve the signal-to-noise ratio. A 9-mm horizontal image containing the fovea was obtained and reinverted for display. Follow-up scans were subsequently compared with previous images to detect subtle changes. Central foveal thickness (CFT) was measured at the centre of the fovea from the inner limiting membrane to the retinal pigment epithelium (RPE). Furthermore, the choroidal thickness (CT) was measured in three different sections from the RPE/Bruch reflective complex to the sclerochoroidal interface by the enhanced depth imaging (EDI) technique, including the subfoveal choroidal thickness (SFCT) and CT of the temporal and nasal regions 1 mm from the fovea (Fig. 1). All OCT scans were independently reviewed and quantitatively measured by the same specialized doctors, each of whom measured each sample three times and calculated the average value. We then determined and used the average values obtained by the two doctors as our final data.

All patients underwent CPS or FLACS with foldable intraocular lens (IOL) implantation. The selection of surgical method was based on the patient’s wishes. FLACS was performed using a femtosecond laser (Lensx, Alcon Laboratories, Inc.). A femtosecond laser was used to perform corneal incisions, capsulotomy, and lens fragmentation with or without astigmatic keratotomy. After femtosecond laser treatment, the patient was transferred to the operating theatre for cataract extraction. The main incision was made at 11:00 and was 2.4 mm in size. Phacoemulsification was performed with a gravity-fluidics torsional phacoemulsification machine (Infiniti, Alcon Laboratories (UK), Itd.). Patients who underwent CPS were prepared for surgery in the same way as those in the laser arm. Intraoperative and postoperative complications were recorded. All surgeries were performed at a single centre by three surgeons (L. L., HP. Z., and S. C.) had more than 10 years of surgical experience and approximately 10,000 cataract operations. The surgeons had also completed at least 100 femtosecond laser–assisted surgeries and used their dominant hand during surgery. The IOL calculation formula was the SRK/T formula, which was corrected using the Wang–Koch eye axis adjustment method: Modified SRK/T optimized AL = 0.8453×(measured AL)+4.0773. The postoperation treatment of the patients were routine levofloxacin, corticosteroids and non-steroidal anti-inflammatory drug (NSAID) eye drugs for two weeks.

The clinical data of all eligible patients and intraoperative and postoperative complications were recorded. Baseline information was also recorded, including age, sex, operative eye, BCVA, IOP, AL, and cataract type (lens opacity classification system III, LOCS III grades). Fundus complications, such as lamellar and full-thickness macular holes (LMH and FMH), foveal and extrafoveal retinoschisis (RS), and epiretinal membrane (ERM), were also considered and recorded.

### Statistical analysis

All the statistical analyses were performed using IBM SPSS Statistics version 22 (IBM Corp., Armonk, NY, USA). Continuous variables are presented as the mean ± standard deviation (SD). Categorical variables are presented as numbers and percentages. Chi-square tests and t tests were used for comparisons between different groups. The main effect, time effect and interaction effect of treatment therapy were analysed by a general linear model. If there was an interaction effect, the independent effect was used for further analysis, and the least significant difference (LSD) test was used for pound-for-pair comparisons. Univariate and multivariate linear regression analysis were used for association analyses. Statistical significance was set at *P* < 0.05. All tests were two-sided.

## Results

### Baseline characteristics

One hundred and two eyes of 102 patients (55 males and 47 females) were included in this study. Fifty-four eyes underwent conventional phacoemulsification surgery (CPS group), whereas the other 48 eyes underwent femtosecond laser-assisted cataract surgery (FLAPCS group). The follow-up time was at least six months. The mean AL of the operated eye was 29.84 ± 2.27 mm, and the maximum AL was 33.35 mm. Patients with extremely high myopia (AL > 30 mm) accounted for 14.7% of the sample. Among these eyes, nuclear cataracts were the main cataract type, occurring in 68 eyes (66.7%). The baseline characteristics are presented in Table 1. The demographic and ophthalmological baseline characteristics of the two treatment groups did not significantly differ (*P* > 0.05).

**Table 1:** Demographic and baseline characteristics of high myopic patients

	Total *n* = 102	CPS group *n* = 54	FLACS group *n* = 48	*P*
Age (years)				0.19
Mean ± SD	56.14 ± 13.66	60.57 ± 11.43	57.09 ± 13.18	
Range	27–85	34–81	27–85	
Sex, n (%)				0.86
Male	55 (53.9%)	29 (53.7%)	26 (54.2%)	
Female	47 (46.1%)	25(46.3%)	22 (45.8%)	
Operated eye				0.98
Right	49 (48.0%)	26 (48.1%)	23(47.9%)	
Left	53(52.0%)	28 (51.9%)	25(52.1%)	
logMAR BCVA	1.18 ± 0.51	1.21 ± 0.45	1.07 ± 0.52	0.17
AL (mm)				0.86
Mean ± SD	29.84 ± 2.27	29.88 ± 2.33	29.49 ± 2.15	
Range	26.05–33.35	26.05–33.35	26.16–32.31	
26–30 mm	87(85.3%)	45(83.3%)	42(87.5%)	
>30 mm	15(14.7%)	9 (16.7%)	6 (12.5%)	
Cataract type				0.61
Nuclear	68(66.7%)	34(63.0%)	34(70.8%)	
Cortical	6(5.9%)	4(7.4%)	2(4.2%)	
Posterior subcapsular	28(27.5%)	16(29.6%)	12(25.0%)	

SD, standard deviation; BCVA, best-corrected visual acuity; AL, axial length; CPS group, conventional phacoemulsification; FLACS group, femtosecond laser-assisted phacoemulsification

### Changes in BCVA within and between groups

The logMAR BCVA did not significantly differ between the two groups at baseline (*P* = 0.171). A general linear model showed that the main effect and time effect of treatment were significantly different for logMAR BCVA (F_main_= 159.97, F_time_ =321.35, both *P* < 0. 001), but there was no significant difference in the interaction effect (F_interaction_= 9.59, *P = 0.1)*. Further comparisons within groups revealed that one week and six months after surgery, the logMAR BCVA of both groups improved significantly compared with that before surgery (both *P* < 0.001), and there was no significant difference between one week and six months after surgery (*P* = 0. 888 and 0.647). Comparisons between groups revealed that the mean improvements in logMAR BCVA (preoperative minus month 6) were 0.77 ± 0.36 and 0.82 ± 0.47 in the CPS group and the FLACS group, respectively. However, no difference was detected in BCVA improvement between the groups (*P* = 0.554).

### Changes in OCT parameters within and between groups

Tables 2 and 3 display the pre- and postoperative OCT parameters within and between groups. The baseline CFT and CT of the four regions did not significantly differ between the two groups and were comparable (all *P* > 0.05). The time and interaction effect of treatment were not significantly different for CFT (F_time_ =1.12, *P =* 0. 294, F_interaction_= 0.509, *P =* 0.601). There was a slight increase in CFT at one week postoperatively, but CFT became thinner at six months postoperatively than at baseline in both groups. However, these changes did not present statistically significant differences compared to the preoperative values (all *P* > 0.05). Comparisons between groups revealed no significant differences in the CFT change between the two groups at any postoperative time point (all *P* > 0.05). The time effect of treatment was not significantly different among the three CT regions (F_time_= 3.52, 2.48, 3.39, *P* = 0.061, 0.105, 0.069). Comparisons within groups: CT of the three regions decreased at one week postoperatively, and they continued to decrease at six months postoperatively, although these changes were not statistically significant compared to the preoperative values (all *P*> 0.05). The interaction effect of treatment was significant for SFCT and temporal 1 mm CT (F_interaction_= 4.72 and 4.28, *P =* 0.011 and 0.017), but there was no significant difference for nasal 1 mm CT (F_interaction_= 1.64, *P =* 0.204). Further comparisons between groups revealed that the SFCT in the CPS group was dramatically lower than that in the FLACS group at one week and six months postoperatively (*P* = 0.03 and 0.026). The temporal CT of 1 mm in the CPS group was significantly lower than that in the FLACS group at one week postoperatively (*P* = 0.046), but there was no significant difference at six months postoperatively (*P* = 0.079).

**Table 2:** Comparison between the Log MAR BCVA and OCT parameters of the two groups

Time/ Parameter (Mean ± SD)	CPS group	FLACS group		*P*
Preoperative				
CFT (µm)	205.67 ± 72.29	223.17 ± 47.26		0.204
SFCT	68.29 ± 43.64	82.64 ± 38.32		0.113
Nasal 1 mm CT (µm)	61.48 ± 47.64	75.14 ± 42.38		0.263
Temporal 1 mm CT (µm)	70.87 ± 58.05	81.94 ± 45.27		0.337
Postoperative 1 week				
CFT (µm)	210.89 ± 74.48	224.66 ± 48.15		0.329
SFCT (µm)	62.43 ± 48.46	82.28 ± 61.12		0.03*
Nasal 1 mm CT (µm)	57.96 ± 41.11	72.53 ± 42.36		0.058
Temporal 1 mm CT(µm)	64.29 ± 58.89	88.47 ± 50.45		0.046*
Postoperative 6 months				
CFT(µm)	203.59 ± 76.23	220.86 ± 50.01		0.234
SFCT (µm)	58.70 ± 44.62	80.36 ± 44.16		0.026*
Nasal 1 mm CT (µm)	55.39 ± 39.63	71.31 ± 40.22		0.067
Temporal 1 mm CT (µm)	60.96 ± 50.32	79.47 ± 45.28		0.079

OCT, optical coherence tomography; CFT, central foveal thickness; SFCT, subfoveal choroidal thickness; CT, choroidal thicknes

**Table 3:** Change of log MAR BCVA and OCT parameters in high-myopia eyes over time

	CPS group (mean ± SD)			*P*_A1_	*P*_A3_	FLACS group (mean ± SD)			*P*_B1_	*P*_B3_
Time	Preoperative	Postoperative	Postoperative			Preoperative	Postoperative	Postoperative		
		one week	six months				one week	six months		
Log MAR BCVA	1.21 ± 0.45	0.48 ± 0.31	0.45 ± 0.28	0.000	0.000	1.07 ± 0.52	0.26 ± 0.36	0.25 ± 0.34	0.000	0.000
CFT (µm)	205.67 ± 72.29	210.89 ± 74.48	203.59 ± 76.23	0.716	0.885	223.17 ± 47.26	224.66 ± 48.15	220.86 ± 50.01	0.896	0.841
SFCT (µm)	68.29 ± 43.64	62.43 ± 48.46	58.70 ± 44.62	0.505	0.276	82.64 ± 38.32	82.28 ± 61.12	80.36 ± 44.16	0.4	0.825
Nasal 1 mm CT (µm)	61.48 ± 47.64	57.96 ± 41.11	55.39 ± 39.63	0.671	0.462	75.14 ± 42.38	72.53 ± 42.36	71.31 ± 40.22	0.791	0.901
Temporal 1 mm CT(µm)	70.87 ± 58.05	64.29 ± 58.89	60.96 ± 50.32	0.542	0.385	88.47 ± 50.45	88.28 ± 66.86	79.47 ± 45.28	0.556	0.824

*P*_*A1*_, *P*_*A3*_ : the comparison between pre- and one week and six months postoperative in CPS group, respectively. *P*_*B1*_, *P*_*B3*_ : the comparison between pre- and one week and six months postoperative in FLACS group, respectively

BCVA, best-corrected visual acuity; OCT, optical coherence tomography; CPS group, conventional phacoemulsification; FLACS group, femtosecond laser-assisted phacoemulsification. CFT, central foveal thickness; SFCT, subfoveal choroidal thickness; CT, choroidal thicknes

### Factors influencing postoperative BCVA

**Fig. 2:**
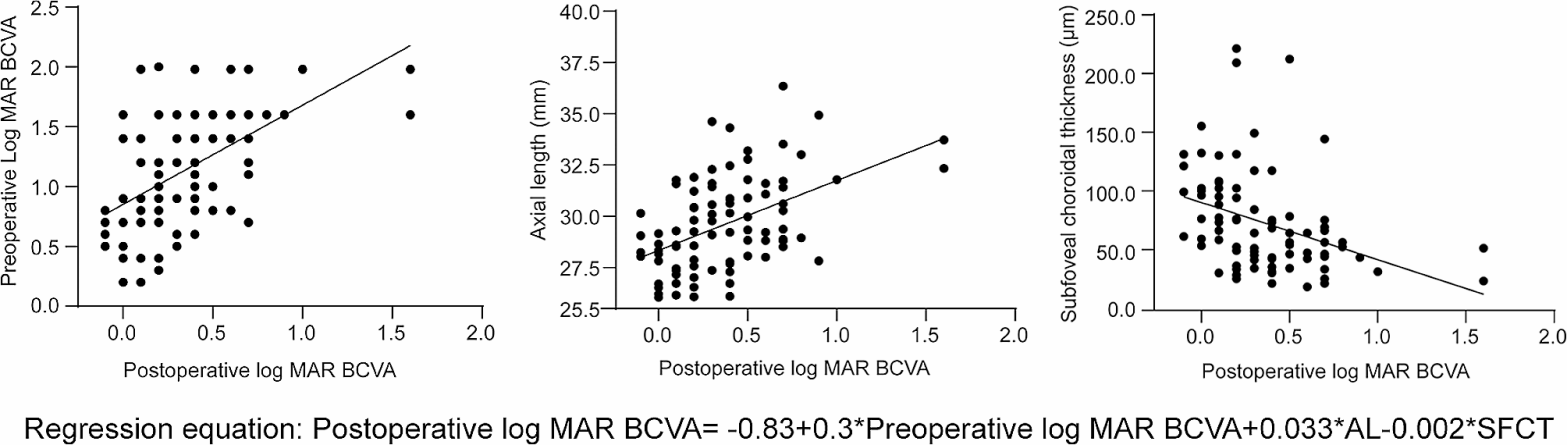
Multivariate linear regression analysis model for the identification of risk factors for postoperative logMAR BCVA with preoperative logMAR BCVA (**A**), axial length (**B**), and subfoveal choroidal thickness (**C**). The regression equation showed that the postoperative logMAR BCVA was positively correlated with the preoperative logMAR BCVA and AL but negatively correlated with the SFCF

Table 4 summarizes the results of the univariate and multivariate linear regression analyses that identified the influencing factors for logMAR BCVA at six months postoperatively. According to the univariate model, AL, preoperative logMAR BCVA, and CT of the three regions were strongly correlated with postoperative BCVA (all *p* < 0.05). However, only the AL, preoperative logMAR BCVA and SFCT remained significant in the multivariate model. Postoperative logMAR BCVA revealed a positive correlation with preoperative logMAR BCVA and AL but a negative correlation with SFCT (Fig. 2).

**Table 4:** Univariate and multivariate linear regression analysis for the identification of risk factors of log MAR BCVA at 6 months postoperative

Baseline Variables	Univariable analysis			Multivariable analysis (Method: Backward)		
	coefficient	95% CI	*P*	coefficient	95% CI	*P*
Axial length	0.068	0.042∼0.095	< 0.001	0.033	0.006∼0.06	0.016
log MAR BCVA	0.364	0.247∼0.481	< 0.001	0.300	0.188∼0.412	< 0.001
CFT (µm)	-8.92*10^− 5^	-0.001∼-0.001	0.869			
SFCT (µm)	-0.003	-0.004∼-0.001	< 0.001	-0.002	-0.003∼-0.00	0.016
Nasal 1 mm CT (µm)	-0.003	-0.004∼-0.002	< 0.001			
Temporal 1 mm CT (µm)	-0.002	-0.003∼-0.001	0.001			

BCVA, best-corrected visual acuity; CFT, central foveal thickness; SFCT, subfoveal choroidal thickness; CT, choroidal thicknes

### Complications of cataract surgery in high myopia eyes

Intraoperative complications included posterior capsular rupture (PCR) in two eyes (1.9%), both of which occurred in the conventional phacoemulsification group, and the IOL was implanted in the ciliary groove. Four eyes out of 102 (3.9%) developed postoperative complications during the follow-up period, including rhegmatogenous retinal detachment (RRD) in two eyes after 6 months and 19 months and vitreous haemorrhage due to retinal breaks in one eye after two months. Vitrectomy was performed in all patients with postoperative complications. Additionally, one eye developed LHM three months postoperatively, and the patient was excluded. All these complications occurred in eyes with extremely high myopia. Transiently increased IOP was observed in 10 eyes on the first day after the operation, and the IOP returned to normal one week postoperatively. The IOP of all eyes remained stable throughout the study. No myopic retinoschisis progression or endophthalmitis occurred during the follow-up period.

## Discussion

Subclinical macular oedema after cataract surgery has attracted substantial attention from surgeons due to the potential hazard of this complication to vision outcomes [9]. Nuclear cataracts accounted for the largest proportion and structural characteristics of highly myopic eyes, especially in patients with high myopia cataracts, both of which increase the difficulty of cataract surgery. Thus, it is very difficult to estimate postoperative visual outcomes in these eyes. The photoreceptor cells in the macula, which consists of the most important layer of the retina, are fed through the choroidal vessels. Choroidal thinning in myopia is well established and is associated with the progression of myopic macular degeneration [17].

CPS is the most routine operation in health care worldwide and has generally improved the outcome of cataract surgery, but certain postoperative complications in the retina may still occur and are associated with worse postoperative visual outcomes. Moreover, femtosecond laser cataract surgery is an advanced technique that has been shown to potentially reduce complications and improve visual outcomes by enabling more precise corneal incisions, capsular centration, and lens fragmentation than CPS [18]. However, the suction ring used during femtosecond laser treatment causes a temporary increase in IOP, which may induce various microstructural changes in the retina and choroid and ocular blood flow, especially in high-myopia cataract patients [19, 20].

Our data revealed that these two technologies are effective and safe for treating highly myopic cataract patients. However, there were no significant differences in visual improvement or complications between femtosecond laser and phacoemulsification techniques for cataract surgery in highly myopic eyes. Previous studies obtained different results in comparisons of CPS and FLACS. Some investigations achieved better visual acuity outcomes after FLACS than after CPS [21, 22], whereas others detected no significant differences between the outcomes of the two surgical approaches [23, 24]. To determine the factors influencing postoperative BCVA in high myopic cataract eyes, we performed logistic regression analysis and revealed that the AL, preoperative BCVA and CT of different regions rather than the CFT were strongly correlated with postoperative BCVA according to univariate logistic regression analysis. However, only eyes with a longer AL, worse preoperative BCVA and thinner SFCT were significantly associated with postoperative BCVA according to multivariate analysis. The results were in line with the results of a hospital-based prospective cohort study included in the Shanghai High Myopia Study [13]. We speculated that this may be due to sight-threatening macular complications such as foveal RS and severe choroidal atrophy with the elongation of the AL. Ye et al. [25] reported that the myoid and ellipsoid zone thickness was affected by CT and was associated with BCVA, which could explain the relationship between CT and BCVA.

In our investigation, we found a slight increase in CFT based on OCT at one week postoperatively, but CFT became thinner at 6 months postoperatively than preoperatively in both groups, but the change was not clinically significant. However, Appolloni et al. [26] compared the different impact on choroid structure between CPS and FLACS, both groups displayed a significant increase in CFT, SFCT, and choroidal vascular index (CVI), but at the follow-up visit of 1 month, all OCT parameters did not display any significant difference. İçöz M et al. [27] evaluated structural and vascular changes in the choroid at first and third month after surgery in 50 patients and reported an increase of choroidal thickness at the first postoperative month and reached the preoperative values at the third month, and CVI increased at the first and third postoperative months. It was clear from the previous studies and our sutdy that despite the early effects of surgery on the retina and choroid varied, the long-term outcomes were not significantly different from those before treatment. In addition, we also found that CT of the different regions decreased as time progressed after surgery, but there was no significant change in either group. Recent research by Zhao L et al. [28] revealed that the SFCT at 1 week and 1 month was significantly greater than that before surgery in both groups, but the SFCT decreased at 3 months after surgery compared with that at 1 month after surgery. However, these findings contrast with our results. Although the factors that affect the thickness of the SFCT after cataract surgery are still unknown, they are speculated to be related to many factors. On the one hand, surgical manipulation causes significant release of inflammatory mediators, and the inflammatory cascade impairs the blood–aqueous and blood–retinal barriers and promotes vascular permeability, which may contribute to the thickness of the retina and choroid [29]. On the other hand, the previous researches showed that high myopia may be a risk factor for early IOP elevation after uneventful cataract surgery [30]. The increase in IOP during the operation leads to a decrease in ocular perfusion pressure, which leads to temporary thinning of the choroid [31]. It is not clear whether these changes affect choroidal remodelling. Especially in patients with high myopia, the rigidity of the eyeball is poor, and this effect may be more lasting. The limitations of this study include the small sample size and short observation time, and the results must be further verified. However, whether the subsequent CT would continue to decrease remains to be determined. Moreover, nuclear cataracts are more predominant in high myopia. We speculate that the femtosecond laser enables lens fragmentation, which reduces the phacoemulsification power and phaco time during operation and may be a crucial factor contributing to this change.

In terms of macular changes between CPS and FLACS, we did not find any difference between the two groups when comparing the CFT at one week or six months after surgery. A previous study also confirmed the absence of a difference in the mean change in CFT between the CPS and FLACS [23], which was similar to our results. However, the SFCT was significantly lower in the CPS group. We hypothesize that this is related to preoperative choroidal thickness rather than surgical procedures. The choroid thickness of the CPS group was thinner than that of the FLACS group at the baseline. Though the difference had no statistical significance, the thinner baseline choroid brought less blood flow, which means high myopia patients are more susceptible to the influence of intraoperative IOP fluctuations [32]. And the length of the intraocular perfusion time in surgery is proportionate to the choroidal remodeling after surgery in highly myopia patients which may occur immediately during surgery and continues to develop in the long run. Earlier evidence also showed that retinal sensitivity and visual acuity had a greater correlation with CT but were not correlated with CFT in highly myopic patients [33]. These findings are consistent with the evidence that in some cases of retinal atrophy combined with ERM, the CFT is thickened, but the SFCT is still thinner.

Two eyes (1.9%) in the CPS group underwent intraoperative PCR. A previous meta-analysis reported that the incidence of PCR during phacoemulsification ranged from 1.8 to 15.6% [11]. The rate of PCR in our study was in line with the results of previous meta-analyses but at a relatively lower limit (1.9%), which might have resulted from the use of advanced surgical devices and the fact that the procedures were performed by more experienced surgeons. High myopia is also a risk factor for spontaneous RD, and earlier studies revealed that the prevalence of RD after phacoemulsification was 2.8% in high myopia patients and 0.4% in emmetropes; AL was the most significant risk factor for RD [34, 35]. Another investigation established that the postcataract surgery risk of RD patients was 2.74- to 18.90-fold greater than that of nonmyopia patients [36]. According to our study, 1.9% of the highly myopic eyes suffered from RD, and a new retinal tear occurred in one eye; all of these cases occurred in patients with AL > 30 mm, which was consistent with the results of the aforementioned clinical studies. Therefore, regular fundal examination, which can prevent the occurrence of serious retinal complications, is particularly important for cataract patients with high myopia.

### Limitations

The main limitation of this study is its retrospective design, which might have added a potential selection bias. In addition, the various follow-up times employed might have led to follow-up bias. Furthermore, these surgeries were performed by three different surgeons but may have had a slight effect on the CFT and/or CT. However, the relatively young age of the patients and the elimination of major confounding risk factors, such as diabetes, hypertension, and glaucoma, are among the advantages of our study. Our present findings are valuable as a preliminary investigation. Future prospective randomized clinical trials with continuous follow-up should further estimate the changes in retinal and choroidal thickness and compare them between PCS and FLACS in myopic patients.

## Conclusion

The results obtained in our study suggest that FLACS does not provide an additional BCVA benefit for myopic patients over CPS. The influencing factors of postoperative BCVA included preoperative logMAR BCVA, AL, and SFCT. Notably, CPS had a positive effect on SFCT, but no effect was exerted on CFT. However, FLACS had less impact on either CFT or CT. With the extension of the axial length, the thickness of the choroid decreased, which was correlated with worse postoperative BCVA.

## Data Availability

No datasets were generated or analysed during the current study.

## References

[1] Grzybowski A, Kanclerz P, Tsubota K, Lanca C, Saw SM (2020). A review on the epidemiology of myopia in school children worldwide. BMC Ophthalmol.

[2] Baird PN, Saw SM, Lanca C, Guggenheim JA, Smith Iii EL, Zhou X (2020). Myopia Nat Rev Dis Primers.

[3] Holden BA, Fricke TR, Wilson DA, Jong M, Naidoo KS, Sankaridurg P (2016). Global prevalence of myopia and high myopia and temporal trends from 2000 through 2050. Ophthalmology.

[4] Cho BJ, Shin JY, Yu HG (2016). Complications of pathologic myopia. Eye Contact Lens.

[5] Zhu X, Li D, Du Y, He W, Lu Y (2018). DNA hypermethylation-mediated downregulation of antioxidant genes contributes to the early onset of cataracts in highly myopic eyes. Redox Biol.

[6] Kanthan GL, Mitchell P, Rochtchina E, Cumming RG, Wang JJ (2014). Myopia and the long-term incidence of cataract and cataract surgery: the Blue mountains Eye Study. Clin Exp Ophthalmol.

[7] Zhu XJ, Zhou P, Zhang KK, Yang J, Luo Y, Lu Y (2013). Epigenetic regulation of αA-crystallin in high myopia-induced dark nuclear cataract. PLoS ONE.

[8] Hejsek L, Kadlecova J, Sin M, Velika V, Jiraskova N (2019). Intraoperative intraocular pressure fluctuation during standard phacoemulsification in real human patients. Biomed Pap Med Fac Univ Palacky Olomouc Czech Repub.

[9] Wang Y, Du J, Yang M, Xu Y, Guan H, Wu J (2018). Distinct macular thickness changes after femtosecond laser-assisted cataract surgery of age-related cataract and myopia with cataract. Sci Rep.

[10] Moon JY, Garg I, Cui Y, Katz R, Zhu Y, Le R (2023). Wide-field swept-source optical coherence tomography angiography in the assessment of retinal microvasculature and choroidal thickness in patients with myopia. Br J Ophthalmol.

[11] Yao Y, Lu Q, Wei L, Cheng K, Lu Y, Zhu X (2021). Efficacy and complications of cataract surgery in high myopia. J Cataract Refract Surg.

[12] Mantopoulos D, Prenner JL, Patel VK, Roth DB, Shah SP, Tsavaris O (2021). The effect of elective cataract extraction extraction by phacoemulsification in eyes with vitreomacular traction syndrome. Retina.

[13] He W, Yao Y, Zhang K, Du Y, Qi J, Zhang Y (2022). Clinical characteristics and early visual outcomes of highly myopic cataract eyes: the Shanghai High Myopia Study. Front Med (Lausanne).

[14] Cetinkaya S, Acir NO, Cetinkaya YF, Dadaci Z, Yener Hİ, Saglam F (2015). Phacoemulsificatıon in eyes wıth cataract and high myopia. Arq Bras Oftalmol.

[15] Ashraf H, Koohestani S, Nowroozzadeh MH (2018). Early Macular Changes after phacoemulsification in eyes with high myopia. J Ophthalmic Vis Res.

[16] Lange C, Feltgen N, Junker B, Schulze-Bonsel K, Bach M (2009). Resolving the clinical acuity categories hand motion and counting fingers using the Freiburg Visual Acuity Test (FrACT). Graefes Arch Clin Exp Ophthalmol.

[17] Hoseini-Yazdi H, Vincent SJ, Collins MJ, Read SA, Alonso-Caneiro D (2019). Wide-field choroidal thickness in myopes and emmetropes. Sci Rep.

[18] Sun H, Fritz A, Dröge G, Neuhann T, Bille JF, Bille JF (2019). Femtosecond-Laser-assisted cataract surgery (FLACS). 2019 Aug 14. High resolution imaging in Microscopy and Ophthalmology: New frontiers in Biomedical Optics.

[19] Conway ML, Wevill M, Benavente-Perez A, Hosking SL (2010). Ocular blood-flow hemodynamics before and after application of a laser in situ keratomileusis ring. J Cataract Refract Surg.

[20] Davis RM, Evangelista JA (2007). Ocular structure changes during vacuum by the Hansatome microkeratome suction ring. J Refract Surg.

[21] Kanellopoulos AJ, Asimellis G (2016). Standard manual capsulorhexis / ultrasound phacoemulsification compared to femtosecond laser-assisted capsulorhexis and lens fragmentation in clear cornea small incision cataract surgery. Eye Vis (Lond).

[22] Chee SP, Yang Y, Ti SE (2015). Clinical outcomes in the first two years of femtosecond laser-assisted cataract surgery. Am J Ophthalmol.

[23] Roberts HW, Wagh VK, Sullivan DL, Hidzheva P, Detesan DI, Heemraz BS (2019). A randomized controlled trial comparing femtosecond laser-assisted cataract surgery versus conventional phacoemulsification surgery. J Cataract Refract Surg.

[24] Schweitzer C, Brezin A, Cochener B, Monnet D, Germain C, Roseng S (2020). Femtosecond laser-assisted versus phacoemulsification cataract surgery (FEMCAT): a multicentre participant-masked randomised superiority and cost-effectiveness trial. Lancet.

[25] Ye J, Shen M, Huang S, Fan Y, Yao A, Pan C (2019). Visual acuity in pathological myopia is correlated with the photoreceptor myoid and Ellipsoid Zone Thickness and affected by Choroid Thickness. Invest Ophthalmol Vis Sci.

[26] Appolloni R, Viggiano P, Carrella ML, Evangelista F, Appolloni A, Toto L, Mastropasqua L (2022). Femto-assisted versus conventional phacoemulsification differently impact on choroid structure after surgery. Eur J Ophthalmol.

[27] İçöz M. Evaluation of structural and vascular changes in the choroid after uneventful phacoemulsification surgery. Rom J Ophthalmol. 2023 Jan-Mar;67(1):50–6. 10.22336/rjo.2023.9 PMC10117187 37089807

[28] Zhao L, Tan M, Zhang J et al. Comparative study of FLACS vs conventional phacoemulsification for cataract patients with high myopia. J Cataract Refract Surg Published Online Febr 13, 2024. 10.1097/j.jcrs.0000000000001425 PMC11146191 38350159

[29] Fleissig E, Cohen S, Iglicki M, Goldstein M, Zur D (2018). Changes in choroidal thickness in clinically significant pseudophakic cystoid macular edema. Retina.

[30] He W, Wei L, Liu S, Huang Z, Qi J, Zhang K (2023). Role of Optic nerve head characteristics in Predicting intraocular pressure spikes after cataract surgery in highly myopic eyes. Ophthalmol Ther.

[31] Chen W, Chen H, Mi L, Li J, Lin H, Chen W (2022). Subfoveal Choroidal Thickness after Femtosecond Laser-assisted cataract surgery for age-related cataracts. Front Med (Lausanne).

[32] Guan Huaijin (2021). Paying close attention to the impact of cataract phacoemulsification surgery on ocular tissue. Chin J Exp Ophthalmol.

[33] Zaben A, Zapata MÁ, Garcia-Arumi J (2015). Retinal sensitivity and choroidal thickness in high myopia. Retina.

[34] Al Muammar AR, Al-Harkan D, Al-Rashidy S, Al-Suliman S, Mousa A (2013). Frequency of retinal detachment after cataract surgery in highly myopic patients. Saudi Med J.

[35] Daien V, Le Pape A, Heve D, Carriere I, Villain M, Incidence (2015). Risk factors, and impact of age on retinal detachment after cataract surgery in France: A National Population Study. Ophthalmology.

[36] Qureshi MH, Steel D (2020). Retinal detachment following cataract phacoemulsification-a review of the literature. Eye (Lond).

